# Visual cortical activity in Charles Bonnet syndrome: testing the deafferentation hypothesis

**DOI:** 10.1007/s00415-024-12741-2

**Published:** 2025-02-11

**Authors:** Katrina daSilva Morgan, Daniel Collerton, Michael J. Firbank, Julia Schumacher, Dominic H. ffytche, John-Paul Taylor

**Affiliations:** 1 Translational and Clinical Research Institute, Campus for Ageing and Vitality, Newcastle Upon Tyne, NE4 5PL UK; 2 Deutsches Zentrum Für Neurodegenerative Erkrankungen Standort Rostock/Greifswald, Rostock, Mecklenburg-Vorpommern Germany; 3 Department of Neurology, University Medical Center Rostock, Rostock, Germany; 4 Institute of Psychiatry, Psychology and Neuroscience, King’s College London, De Crespigny Park, London, SE5 8AF UK

**Keywords:** Cortical excitability, Eye disease, Release phenomena, Alpha reactivity, Phosphene threshold

## Abstract

Visual hallucinations in individuals following sight loss (Charles Bonnet syndrome; CBS) have been posited to arise because of spontaneous, compensatory, neural activity in the visual cortex following sensory input loss from the eyes—known as deafferentation. However, neurophysiological investigations of CBS remain limited. We performed a multi-modal investigation comparing visual cortical activity in 19 people with eye disease who experience visual hallucinations (CBS) with 18 people with eye disease without hallucinations (ED-Controls; matched for age and visual acuity) utilising functional MRI, EEG, and transcranial magnetic stimulation (TMS). A pattern of altered visual cortical activity in people with CBS was noted across investigations. Reduced BOLD activation in ventral extrastriate and primary visual cortex, and reduced EEG alpha-reactivity in response to visual stimulation was observed in CBS compared to ED-Controls. The CBS group also demonstrated a shift towards lower frequency band oscillations in the EEG, indicative of cortical slowing, with significantly greater occipital theta power compared to ED-controls. Furthermore, a significant association between reduced activation in response to visual stimulation and increased excitability (in the form of reduced TMS phosphene thresholds) was observed in CBS, indicating persistent visual cortical activation consistent with hyperexcitability, which was found to be significantly associated with increased hallucination severity. These results provide converging lines of evidence to support the role of increased visual cortical excitability in the formation of visual hallucinations in some people following sight loss, consistent with the deafferentation hypothesis.

**Supplementary Information:**

The online version contains supplementary material available at 10.1007/s00415-024-12741-2.

## Introduction

Charles Bonnet syndrome (CBS) is a condition in which people experience visual hallucinations (VH) secondary to visual impairment in the absence of psychiatric illness or cognitive impairment [1]. While some definitions of CBS focus on complex hallucinatory phenomena (e.g., images of people, animals, scenes etc.), VH also include simple phenomena such as flashes of light, geometric patterns and shapes [2, 3]. The precise aetiology of VH and why only some people experience them following sight loss remains uncertain. Reformulating the earlier terminology of release phenomena [4], it has been hypothesised that chronic deafferentation through loss of visual input from the eyes results in compensatory spontaneous hyperexcitability of the visual cortex leading to VH [5].

A limited number of neurophysiological investigations have provided indirect support for altered excitability in CBS. Ffytche et al*. *[6], using functional magnetic resonance imaging (fMRI) in a group of CBS participants observed activation of specific visual areas reflecting the particular content of hallucinations during and immediately prior to VH onset suggesting cortical hyperexcitability. Furthermore, greater visual cortical activity in response to visual stimulation was observed in CBS during periods of non-stimulation compared to eye-disease controls, indicating constant (tonic) background activity in CBS [6].

Investigation of the visual system in CBS using electroencephalography (EEG) observed generalised decreased alpha power in CBS [7], indicative of increased visual cortical excitability [8]. Similarly, significantly elevated visual cortical responses to peripheral field stimulation, as evidenced with steady-state visual evoked potentials (SSVEPs), have been noted in CBS compared to non-hallucinating eye disease controls [9].

Transient increases in visual cortical excitability also occur following short-term visual deprivation in normal sighted individuals, with false perceptions of light (or ‘phosphenes’) being reported in response to lower intensity transcranial magnetic stimulation (TMS) compared to pre-deprivation [10]. Similarly, increased extrastriate and posterior parietal activity on fMRI has been associated with the occurrence of simple hallucinations following periods of blindfolding [11].

Nevertheless, the research into the cortical mechanisms involved in CBS is still substantially lacking, hindering effective intervention development. The neurophysiological investigations in CBS predominantly consist of single or small group case studies (i.e., [7, 12–14]), often only reporting incidental clinical findings. As vision impairment has been associated with changes to the visual cortex, including neuronal degeneration [15, 16], the lack of comparisons to similarly sight-impaired control groups in the literature has meant that it is difficult to establish whether altered activity observed in CBS is simply a general consequence of vision loss or specifically linked to VH formation.

It has previously been speculated that complex hallucinations may result from dysfunction in higher cortical areas, while simple hallucinations are a consequence of posterior occipital dysfunction [17]. Nonetheless, while both simple and complex hallucinatory phenomena have been reported in CBS, differences in cortical activation and excitability between the two, if any, have yet to be investigated.

The aim of the present study was to compare people with eye disease who experience VH (CBS) with age- and vision-matched people with eye disease but no VH (ED-Controls) on measures of visual cortical activity and excitability using a combination of fMRI, EEG and TMS investigative approaches.

If the deafferentation hypothesis is correct, one would predict participants with CBS would demonstrate evidence of increased visual cortical excitability compared to ED-controls across a range of measures including blood oxygen level dependent (BOLD) response to visual stimulation, occipital alpha power and reactivity and occipital phosphene thresholds. Furthermore, if there are differences in the underlying mechanism of simple and complex hallucinations, this might be reflected in differences in visual cortical excitability measures for people with CBS experiencing predominantly simple hallucinators compared to those experiencing predominantly complex hallucinations.

## Materials and methods

Thirty-seven participants (14 males; mean age: 79, SD: 8.55, range: 53–93) were recruited to the study from ophthalmology services across North-East England and from a Macular Society database of members interested in research participation. Of these, 19 participants were diagnosed with CBS following sight loss according to the criteria of Teunisse et al. [1], modified to include both simple and complex hallucinations: i.e. VH in the presence of eye disease sufficient to cause visual impairment, the absence of hallucinations in other modalities, delusions, impaired insight, or concurrent psychiatric or neurodegenerative illness. The participants with a history of moderate-to-severe cerebrovascular disease or epilepsy were also excluded. Following participation in the present study, 16 participants went on to take part in a crossover trial investigating the effects of non-invasive brain stimulation on VH [18]. The remaining 18 participants were diagnosed with eye disease but had never experienced VH during the course of their sight loss (ED-controls) and were matched to the CBS group for age and visual acuity.

Global cognitive function was assessed in all participants prior to participation using the Mini Mental State Exam adapted for blind participants [19] (MMSE-Blind; max score = 27). The inclusion criteria stipulated an MMSE-blind score of ≥ 24 to ensure participants were cognitively intact and did not have dementia. Using G*Power 3.1, a total sample size of 30 was estimated to allow adequate detection of between-group differences with an alpha level of 0.05 at 80% power.

All participants provided written informed consent and ethical approval was granted by the Tyne and Wear South Research Ethics Committee and Newcastle NHS Research and development committees (ref: 17/NE/0131). This study was conducted in concordance with the tenets of the Declaration of Helsinki.

### Neuropsychological assessment

All participants completed a battery of neuropsychological assessments prior to neurophysiological procedures. The presence of VH within the past month was confirmed using the Neuropsychiatric Inventory hallucination subscale [20] (NPI^hall^; max score = 12). This comprises scores for VH frequency (0–4) and severity (0–3) which are multiplied to give an overall hallucination score (0–12; with higher scores indicating more severe hallucinations). Due to its primary use in dementia patients, the NPI contains a caregiver distress scale. However, in the present study, the participants were asked directly about their experiences and therefore the distress scale (0–5) was adapted to indicate the level of distress a participant felt towards their own VH.

The detailed descriptions of VH were collected using an adapted version of the North-East Visual Hallucination Interview [21] (NEVHI); a semi-structured interview designed to investigate VH phenomenology, occurrence, and overall emotional impact. The quantitative scores were assigned to temporal aspects of VH including frequency (1–8) and duration (1–4), and emotional impact including distress (0–10) and irritation (0–10) felt towards/because of VH. Higher scores in all domains denote greater frequency/duration/distress/irritation as rated by participants.

The presence of depressive symptoms was assessed using the 15-item Geriatric Depression Scale [22] (GDS), in which higher scores indicate more depressive symptoms. Independence in day-to-day functioning was assessed using The Instrumental Activities of Daily Living scale [23] (IADL; maximum score = 8).

Binocular visual acuity and contrast sensitivity (utilizing both eyes) were assessed using the computerized Freiburg visual acuity (decimalized Snellen acuity) and contrast test (%) which is a reliable method for evaluating visual function in visually impaired groups [24].

### Neurophysiological assessment

#### Transcranial magnetic stimulation (TMS)

TMS was delivered in a semi-darkened room using a handheld MagStim 70-mm figure-of-eight coil connected to two monophasic Magstim 2002 stimulators via an integrated Bistim2 unit (Magstim Co, Dyfed, Wales). This study used a protocol of paired pulse stimulation of the occipital regions [25, 26]. The phosphene thresholds were assessed in pseudo-random order at up to nine sites cantered over the occiput (10% of nasion–inion distance above the inion) at 2-cm cross-sections. The phosphene threshold was determined utilising a stepwise procedure by increasing stimulation intensity from 60% in 10% increments up to 100% of stimulator output and decreasing intensity in 1% increments if a phosphene was elicited. If phosphenes were elicited at 60%, stimulation intensity was reduced to 30% and followed the same procedure. Four stimulations were given at each intensity, with the lowest intensity required to elicit at least one phosphene defined as the phosphene threshold. The lower threshold (one of four stimulations [*p* = 0.25] opposed to two of four [*p* = 0.50]) was used to minimise the number of participants who failed to respond to TMS [26]. Sham stimulation was randomly interspersed amongst active stimulation (approximately 1:10) to test for accurate reporting.

#### Functional MRI

The participants were scanned on a 3T whole body MR scanner (Achieva scanner; Philips Medical System; The Netherlands) with body coil transmission and a 32-channel head coil receiver.

Functional MRI (fMRI) was collected using the following parameters: FOV = 192; 64 × 64 matrix; 27 slices, 3-mm thick with a 1-mm gap; TR = 1920 ms; TE = 35 ms; 156 volumes. Additional two spin-echo echo planar imaging (EPI) sequences using the same geometry as fMRI were collected with opposing phase encoding directions and used to remove geometric distortion.

#### Visual stimulation task

The majority of participants were expected to have central visual field loss due to macular disease but preserved peripheral vision. As the MRI scanner limits the extent of peripheral vision that can be stimulated, a task was used which was previously piloted in a similar patient group (ffytche et al.; unpublished findings) controlled by the psychophysics toolbox (http://psychtoolbox.org/) extension for MatLab (MathWorks, Natick, Massachusetts, USA). In the first condition, the participants were prompted by a verbal recording to move their gaze from left to right and back across a high contrast checkerboard once every second, stimulating the visual field periphery with each saccade. In the second condition (rest), the participants were asked to look straight ahead while viewing a grey screen (with a verbal prompt repeated every three seconds). Each condition lasted 15-s and was repeated alternately with the entire fMRI task lasting a total of five-minutes. Further description and pilot information relating to the visual task is available in Online Resource 1.

#### Electroencephalography

Focal occipital EEG was recorded using a Starstim 8-channel EEG/tCS data acquisition system (Neuroelectrics, Barcelona, Spain). Seven Ag/AgCl Pi-electrodes (P7, PO7, O1, Oz, O2, PO8, P8) were placed according to the international 10–20 system over occipital and occipital-temporal regions. A single electrode was placed over the left dorsolateral prefrontal cortex (F3). Reference and ground were acquired from the left earlobe and impedances were kept below 5 kΩ. The data were sampled at 500 Hz from DC to 250 Hz. The resting-state activity was recorded over a 5-min period, during alternating eyes-open and eyes-closed conditions so that alertness was maintained at approximately equal levels in both states. The participants were asked to open or close their eyes in 30-s blocks. During the eyes-open condition, the participant was asked to look straight ahead to reduce eye-movement related artefacts and orienting of attention to objects in the environment. Throughout recording, the investigators monitored participants to ensure adherence to the protocol.

### Data processing

#### fMRI

Processing of fMRI data was performed in SPM12 (https://www.fil.ion.ucl.ac.uk/spm/). Data from the two stimulus conditions (eye-movements; rest) were slice time corrected, motion corrected by aligning all functional images together, unwarped using the field map generated from the pair of spin echo EPI images using FSL’s topup script, then co-registered with the T1 anatomical image. Data were transformed into standard space with a voxel size of 3 × 3 × 3 mm, using spatial normalisation parameters from the T1 segmentation, and smoothed using a 6 × 6 × 6 mm full-width at half maximum Gaussian kernel. A high-pass filter of 128-s was used and serial correlations removed using SPM’s AR(1) model.

The initial whole brain analysis of the data was conducted using a general linear model (GLM) in SPM. Comparison of the two conditions was performed by a design matrix which convolved the time course of the conditions with the canonical haemodynamic response function and its first derivative, including motion parameters as covariates. The contrast images were generated from β estimates of this comparison for each participant.

Five regions of interest (ROI) chosen based on previous imaging investigations in similar patient groups (e.g. [6]) were defined in MNI (Montreal Neurological Institute) space averaging across left and right hemispheres: (1) V1 and V2 combined, (2) ventral extrastriate cortex (areas hOC3v & hOC4v)(27), (3) fusiform gyrus, (4) thalamus, (5) precuneus, taken from the SPM Anatomy toolbox (fz-heulich.de/inm/inm-1/DE/Forschung/_docs/SPMAnatomyToolbox/SPMAnatomyTool-box_node.html) [27, 28].

##### EEG

Data pre-processing was performed separately for eyes-open and eyes-closed conditions using the EEGLAB toolbox (version 14) in Matlab. EEG data were bandpass-filtered (1–80 Hz), notch-filtered around 50 Hz, and split into non-overlapping 2-s epochs. Following visual inspection and exclusion of noisy channels and epochs containing gross artefacts, independent component analysis was applied and artefactual components were rejected. Further analysis was then performed on each participant’s first 40 artefact-free 2-s epochs. For each occipital electrode and epoch, power spectral density was computed in Matlab using Bartlett’s method with a Hamming window for frequencies from 2 to 45Hz. The mean power was calculated within standard EEG frequency bands, delta (2–4 Hz), theta (4–5.5 Hz), pre-alpha (5.5–8 Hz), alpha (8–12 Hz), beta (12–30 Hz) and slow gamma (30–45 Hz), averaged across epochs and electrodes, then normalised by total power across the power spectrum. Alpha-reactivity was calculated according to the following formula [29]:

$$\text{alpha reactivity}= \frac{\text{alpha power eyes closed}-\text{alpha power eyes open}}{\text{alpha power eyes closed}}$$

where alpha power was calculated from the O1, Oz and O2 electrodes as the relative power within a frequency bin around the individual alpha peak frequency (± 2Hz). Individual alpha peak frequencies were calculated using the peak in the power spectrum in the extended alpha frequency range (5.5–15 Hz) using eyes-closed data to account for possible alpha slowing in CBS patients. Theta/Alpha ratio, an index used to demonstrate the resting state percentage of alpha versus theta spectral potential [30], and a measure of slowing of the dominant EEG frequency was calculated as:

$${\text{Theta/Alpha}}=\frac{\text{Theta power}}{\text{Theta Power}+\text{Alpha power}}$$

### Statistical analysis

The group differences in neuropsychological and neurophysiological data were examined using one-way ANOVA and Mann–Whitney U tests to establish statistical significance (*p* < 0.05). Where appropriate, an analysis of covariance (ANCOVA) was performed to investigate differences between groups and conditions while controlling for the effects of covariates known to impact the dependent variables (e.g. age). The second-level analysis of fMRI data was performed by comparing contrast images from the two conditions (eye movements—rest) for all participants. The results are shown with a voxelwise threshold of *p* < 0.001 (uncorrected) followed by a clusterwise threshold of *p* < 0.05 family-wise error (FWE)-corrected for multiple comparisons. Exploratory analysis of additional EEG bands (e.g. delta, beta, theta and gamma) and ROI’s not included in the main hypotheses (e.g. fusiform gyrus, thalamus and precuneus), and associations between neurophysiological measures, were corrected for multiple comparisons using the Bonferroni method; uncorrected statistics are reported as *p*_uncorr_. Associations between variables were evaluated using spearman’s correlations.

## Results

### Participant characteristics

Demographic information of participants included in the analysis are shown in Table 1. There were no significant differences between the groups in age, sex, or visual function tested with both eyes open. The majority of participants in both groups were diagnosed with age-related macular degeneration (AMD) in one or both eyes (13/19 CBS, 13/18 ED-control); no participant in either group had complete visual loss (no perception of light) in both eyes at the time of testing. Most CBS participants experienced both simple and complex hallucinations at some point during the course of their eye disease. For further exploratory analysis, CBS participants were divided into two sub-groups according to the type of hallucination they reported most frequently and for the longest duration (on the NEVHI) in the month prior to assessment, with 10/19 reporting predominantly complex VH, and 9/19 predominantly simple VH. Simple hallucinations were defined as phenomena consisting only of simple shapes, colours, lights (photopsia) and lines; complex hallucinations consisted of more detailed and formed phenomena such as visions of people, animals, objects, scenes, or complex patterns. Group assignment also reflected the phenomenology most frequently reported at the time of neurophysiological assessment. Two participants had constant VH that would have been present during investigations. None of the participants with intermittent VH reported them during investigations. GDS scores indicated that neither group had a depressive illness at the time of assessment. The CBS group reported significantly lower IADL scores compared to the ED-controls. No significant differences in age, MMSE scores, visual acuity or contrast were noted between the two CBS sub-groups. The descriptions of hallucination phenomenology and ocular pathology reported by the CBS group can be viewed in Online Resource 2.

**Table 1:** Demographic details of study participants split by primary groups (CBS x ED-Controls) and hallucination sub-group (Complex CBS x Simple CBS)

	CBS	ED-Controls	Sig. (*p*)	Complex CBS	Simple CBS	Sig. (*p*)
*N*	19	18		10	9	
Age	78.74 (± 9.85)	78.94(± 7.21)	.942*	82.90 (± 7.86)	74.89 (± 10.83)	.102
Sex (M:F)	7:12	7:11	1.0**	3:7	4:5	.823**
Years since eye disease diagnosis	11.00 (± 13.95)^a^	5.22 (± 7.65)	.133*	7.22 (± 6.08)	14.78 (± 18.57)	.308
MMSE-Blind (0–27)	25.74(± 1.33)	26.00 (1.14)	.597	25.70 (± 1.25)	25.89 (± 1.27)	.667
GDS (0–15)	2.79 (± 1.27)	2.67 (± 2.54)	.853*	2.70(± 1.06)	2.89 (± 1.54)	.865
IADL (0–8)	6.74 (± 1.10)	7.44 (± 0.78)	.031*	6.80(± 1.14)	6.67(± 1.12)	.767
Visual Acuity	.274 (± .279)	.357 (± .226)	.186	.201(± .238)	.354(± .313)	.269
Visual Contrast (%)	50.03 (± 38.85)^b^	35.66 (± 37.82)	.510	66.83(± 40.14)	28.42(± 25.65)	.063

Data displayed as mean (± standard deviation). Significance was assessed using one-way ANOVA(*) and Mann–Whitney tests dependant on data distribution

*CBS* Charles Bonnet Syndrome, *MMSE* Mini Mental State Examination, *GDS* Geriatric Depression Scale, *IADL* Instrumental Daily Activities of Living scale

^a^*n* = 18 as one participant did not know when they were diagnosed

^b^*n* = 16 as 3 participants did not have enough vision to complete the task

^**^Fisher’s Exact Test

### TMS

**Fig. 1:**
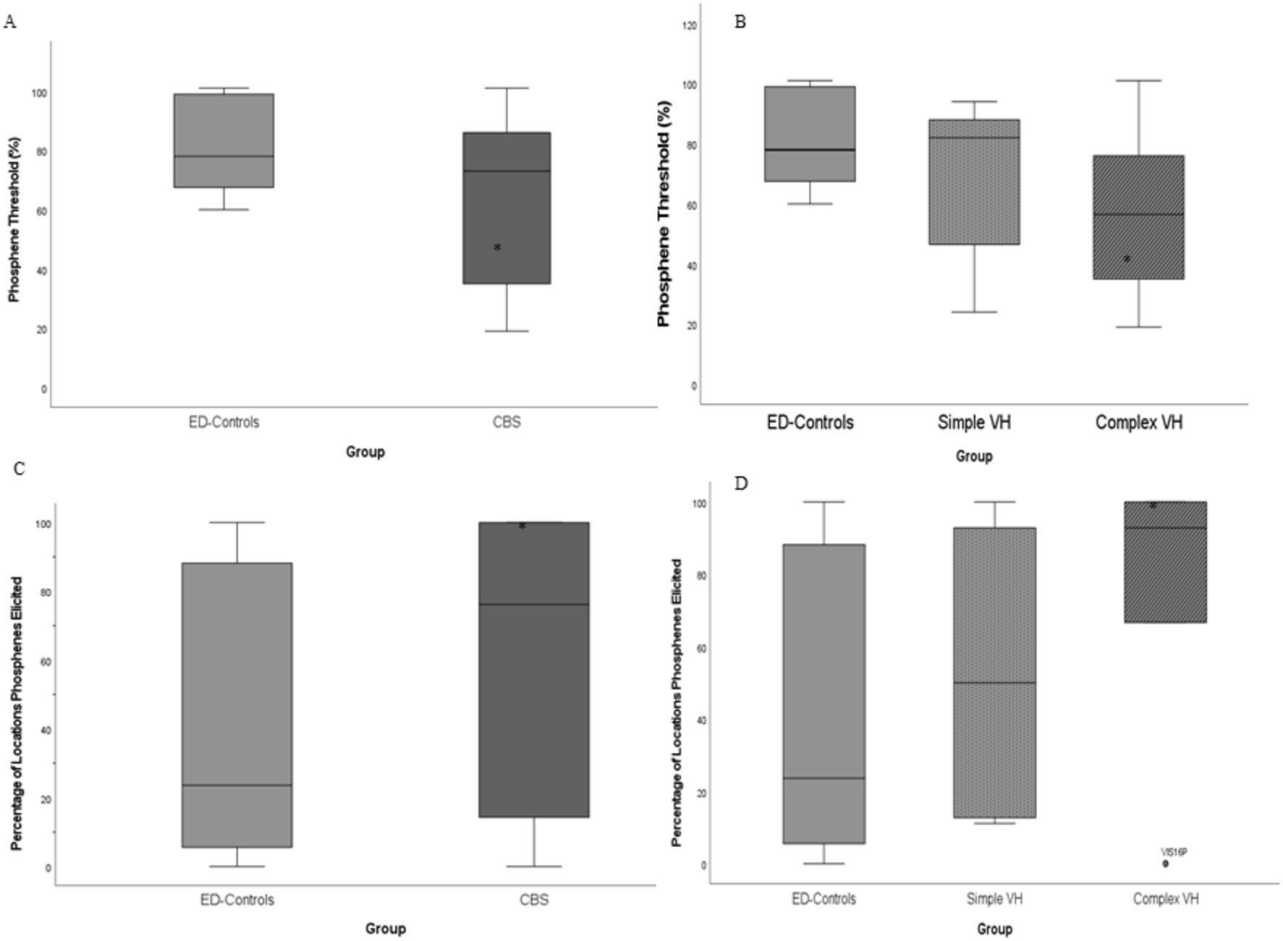
Box Whisker Plots of phosphene threshold and phosphene locations data. Median (line) and standard deviation of phosphene thresholds by group (**A**) and phenomenology sub-groups (**B**) and percentage of locations tested at which phosphenes were elicited by group (**C**) and phenomenology sub-groups (**D**). *denotes participant with continuous hallucinations (*n* = 1). Participant VIS16P (labelled in chart D) was the only member of the CBS group not to report any phosphenes in response to TMS

A total of 14 CBS participants and sixteen ED-controls underwent TMS phosphene testing; however, one ED-Control participant was excluded from analysis due to reporting phosphenes following sham stimulation (final ED-Control *n* = 15). No significant differences in phosphene threshold were observed between CBS (median = 73.00 ± 27.82) and ED-controls (median = 78.00 ± 15.65) groups (z = −1.39, *p* = 0.162). However, the CBS group demonstrated significantly greater variability than ED-controls (Levene’s test; F(1,28) = 9.31, *p* = 0.005; shown in Fig. 1). When split by hallucination subgroup, no significant differences were seen between simple (median = 82.00 ± 26.76) and complex (median = 56.50 ± 30.30) hallucinators, (*z* = −0.77, *p* = 0.452). No significant differences were observed between ED-controls and simple or complex hallucinators (*p* > 0.05*).*

No significant differences in the percentage of locations tested from which phosphenes were elicited were observed between ED-Controls and the CBS group, between complex and simple CBS hallucinators, or simple CBS hallucinators and ED-Controls (*p* > 0.05) (Fig. 1C), and no tendency toward more anterior or posterior locations was noted in any group. However, complex CBS hallucinators were found to report phosphenes from a significantly greater percentage of locations tested compared to ED-controls (*z* = −2.24, *p* = 0.025) (Fig. 1D).

Using participant structural scans, the impact of potential occipital atrophy on TMS field strength was examined by calculating the average distance from the scalp to occipital surface at the occiput in a 1-cm diameter cylinder (method detailed in Taylor et al. [26]). No associations between scalp-occipital surface distance and phosphene thresholds or number of sites at which phosphenes were elicited were observed in any of the groups (*p* > 0.05).

### Phosphene thresholds and VH measures

As age was observed to have a significant association with phosphene thresholds (*r*_s_ = 0.400, *p* = 0.028), Spearman’s partial correlations controlling for age were used. A moderate negative association was observed between phosphene thresholds and VH ratings for NPI severity (*r*_s_ = −0.618, *p* = 0.024), overall NPI^hall^ scores (severity x frequency; *r*_s_ = −0.650, *p* = 0.016) and NEVHI irritation scores (*r*_s_ = −0.559, *p* = 0.047) in the CBS group. No significant associations between VH frequency, duration or distress measured by the NPI or NEVHI, or visual acuity and contrast, and phosphene thresholds were observed in either group (*p* > 0.05).

### fMRI

**Fig. 2:**
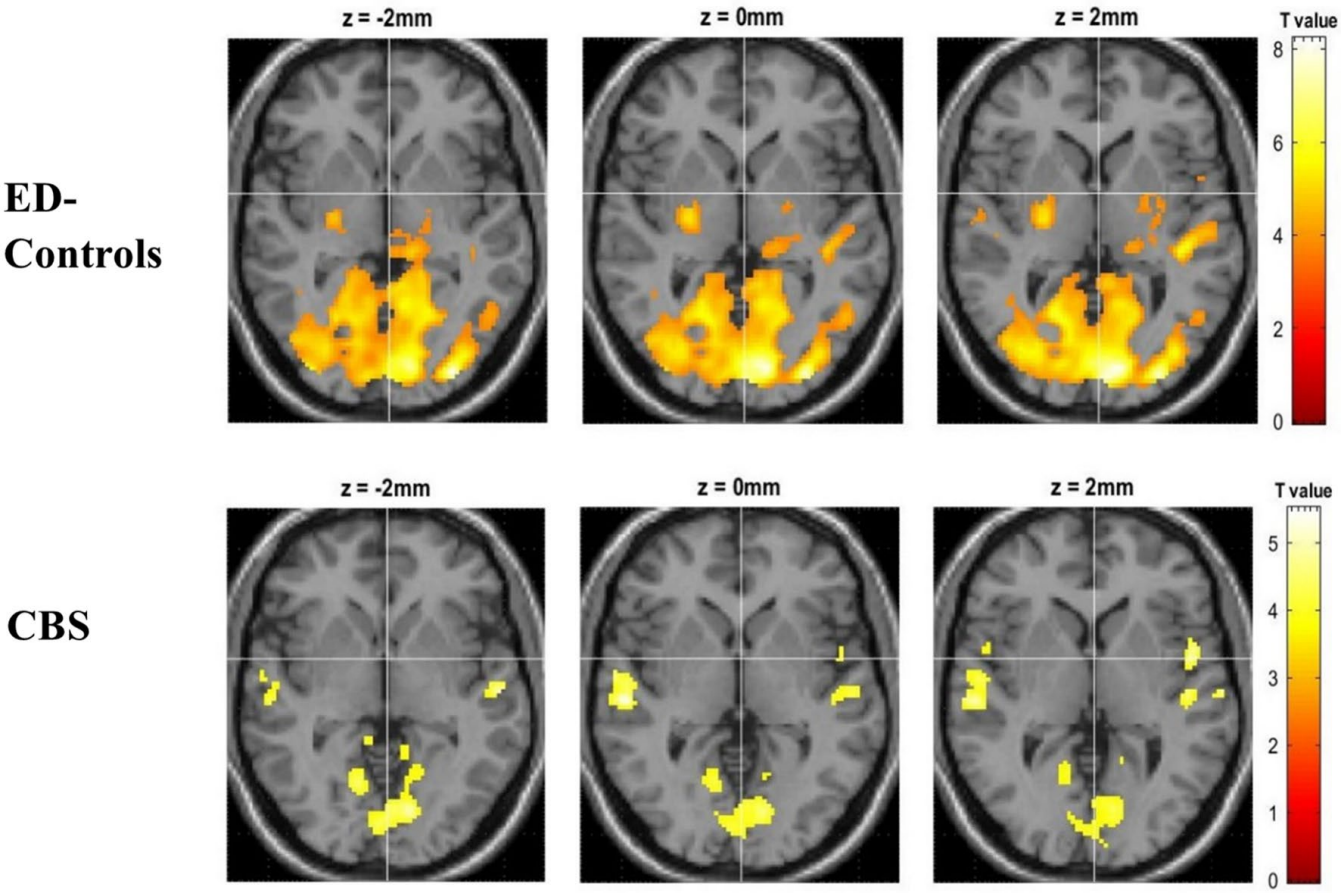
fMRI blood oxygen level dependent activation during an eye-movement task compared to rest by group. Threshold at *p* < 0.001 uncorrected and superimposed on a standard T1 weighted brain image

No significant differences in whole-brain BOLD activation were found between the two (> 0.05-FWE; Fig. 2) despite observable widespread occipito-temporal activation during visual stimulation in ED-controls, compared to only limited activation of the primary visual cortex in the CBS group. Nonetheless, ROI analysis noted significantly reduced ventral extrastriate (t(35) = 2.65, *p* = 0.006) and V1/V2 activity (t(35) = 1.85, *p* = 0.036) in the CBS group compared to ED-controls. Significantly reduced fusiform gyrus (*p*_*uncorr*_ = 0.021) and thalamus (*p*_uncorr_ = 0.036) activities were also noted in CBS compared to ED-controls, but this did not withstand multiple-comparison correction. No significant difference in precuneus activity was noted between groups.

**Fig. 3:**
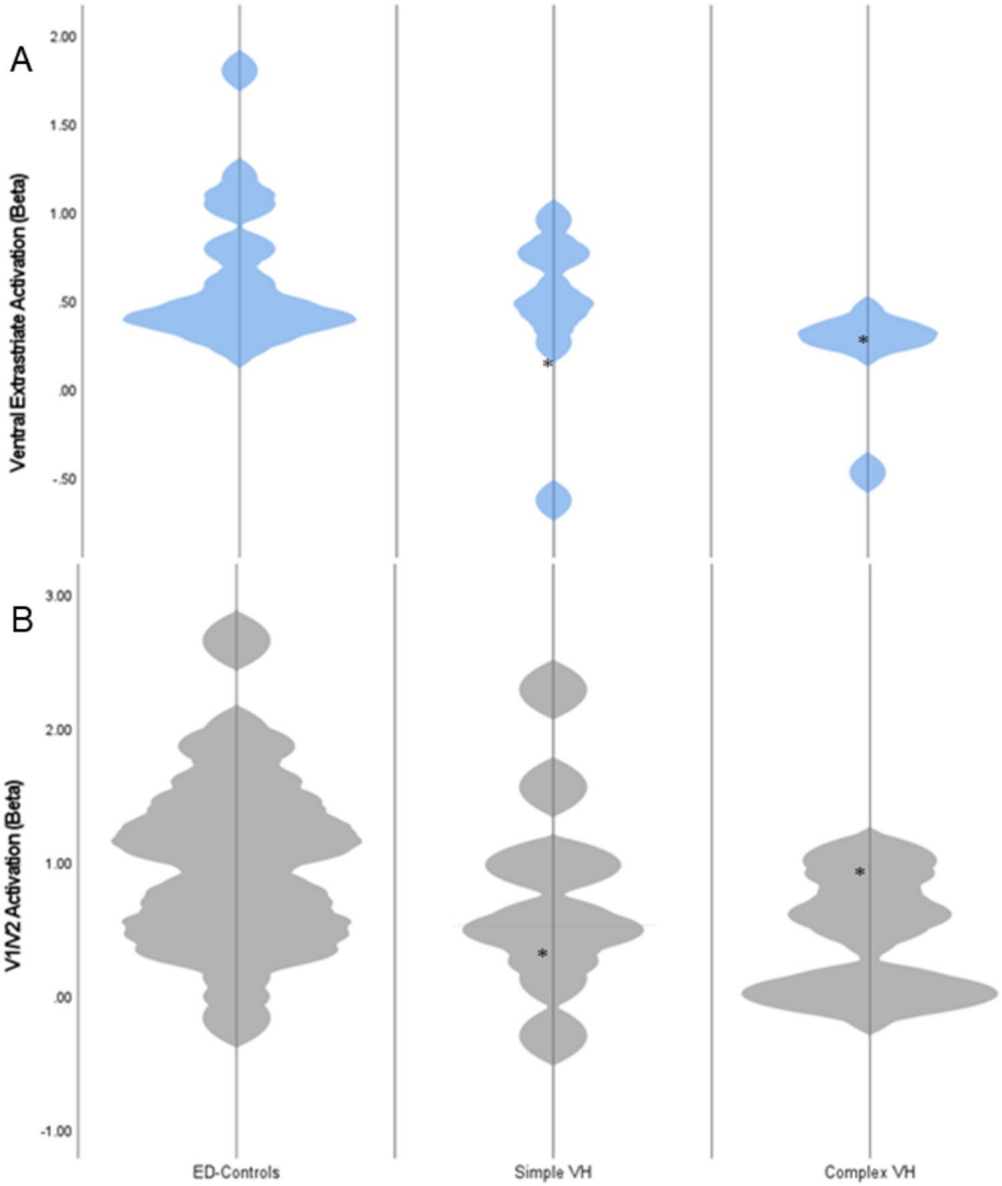
Violin plots comparing numeric distribution of fMRI BOLD activation in ED-controls, CBS simple hallucinators and CBS complex hallucinators in **A** ventral extrastriate and **B** V1/V2. * denotes participants with continuous hallucinations (*n* = 2)

When split by hallucination subgroup, no significant differences in BOLD activity in any of the ROI measured were observed between simple and complex hallucinators, or between simple hallucinators and controls (*p* > 0.05). However, a significant reduction in V1/V2 (z = −2.16, *p* = 0.031) and ventral extrastriate activity (z = −2.88, *p* = 0.003) in complex hallucinators compared to ED-controls was observed (Fig. 3).

### BOLD signal and VH measures

No significant associations between VH ratings on the NPI^hall^ or NEVHI with BOLD activation were observed in the CBS group, regardless of hallucination type (*p* > 0.05).

No association between visual function and BOLD activation was observed across groups.

### EEG

**Fig. 4:**
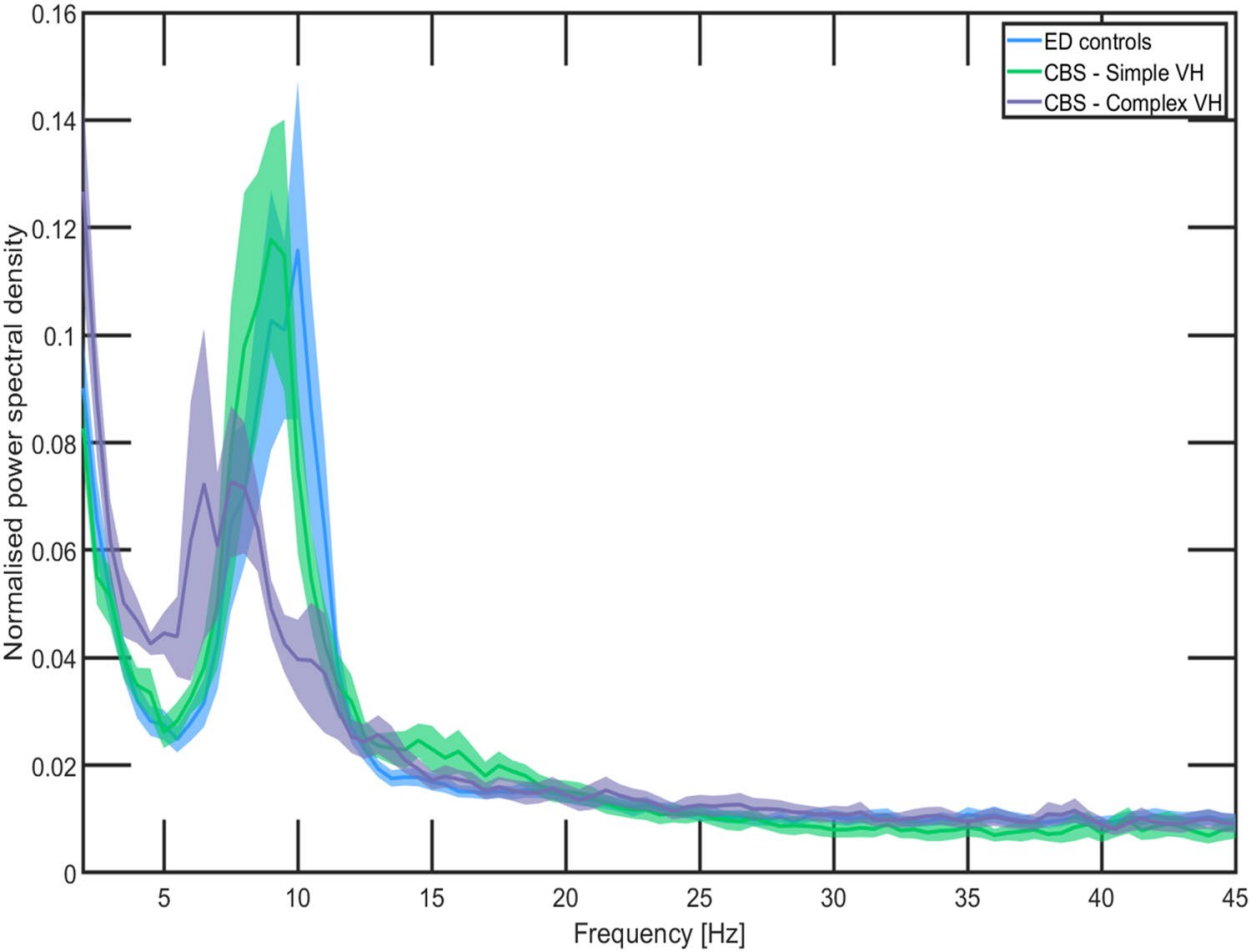
Occipital power spectral density of ED-Controls, CBS Simple hallucinators and CBS Complex hallucinators

An overall shift in the occipital spectral density towards lower frequency band oscillations was observed in the CBS group compared to ED-controls, with a similar pattern demonstrated in complex CBS hallucinators compared to simple (Fig. 4). However, no significant difference in the dominant frequency was observed between each group (Table 2). The CBS group demonstrated significantly greater relative occipital theta power (*z* = −2.50, *p* = 0.012) and decreased alpha-reactivity (*z* = −2.09, *p* = 0.037) compared to ED-controls (Table 2). Similarly, complex CBS hallucinators presented with significantly greater theta power (*z* = −2.57, *p* = 0.009), and theta alpha ratios (*z* = −2.67, *p* = 0.006), but lower alpha power (*z* = −2.40, *p* = 0.016) than simple CBS hallucinators. Complex CBS hallucinators demonstrated significantly greater relative theta power (*z* = −3.26, *p* < 0.001) and theta:alpha ratios (*z* = −2.97, *p* = 0.002), but significantly lower relative alpha power (*z* = −2.49, *p* = 0.012) and alpha-reactivity (*z* = −3.02, *p* = 0.002) compared to ED-controls.

**Table 2:** Median (interquartile range) of relative EEG band power in each group

	Group							
	ED-Controls	CBS (overall)	Simple hallucinators	Complex hallucinators	Sig. (p) ED-controls vs CBS	Sig. (p) Simple vs Complex	Sig. (p) ED vs simple	Sig. (p) ED vs complex
*N*	18	18*	8*	10				
Delta	0.12(.07)	0.13(.06)	0.12(.04)	0.13(.08)	0.372	0.068	0.807	0.109
Theta	0.06(.03)	0.08(.04)	0.06(.03)	0.09(.03)	**0.012**	**0.009**	0.567	** < 0.001**
Pre-Alpha	0.10(.08)	0.11(11)	0.12(.10)	0.11(.13)	0.192	0.897	0.397	0.226
Alpha (eyes closed)	0.26(.27)	0.19(.18)	0.30(.15)	0.15(.09)	0.126	0.**016**	0.892	**0.012**
Beta	0.22(.13)	0.30(.09)	0.30(.09)	0.30(.11)	0.308	0.762	0.311	0.524
Slow Gamma	0.13(.10)	0.11(.09)	0.10(.09)	0.13(.12)	0.584	0.408	0.367	0.981
Theta:Alpha	0.38(037)	0.57(.74)	0.31(.24)	0.90(.57)	0.059	**0.006**	0.935	**0.002**
Alpha Reactivity	0.34(.32)	0.11(.60)	0.49(.70)	0.02(.35)	**0.037**	0.101	0.892	0.**002**
Dominant Frequency	9.49(1.13)	8.88(1.33)	9.34(1.04)	8.68(1.89)	0.074	0.274	0.311	0.072

Significant differences in bold (Wilcoxon signed rank test, *p* < 0.05)

*Data missing from one participant due to equipment failure

### EEG indices and VH measures

No significant associations were observed between VH measures, or visual function, and any EEG band oscillations following multiple comparison corrections.

### Exploratory multi-modal analysis: relationships with visual cortical excitability

**Fig. 5:**
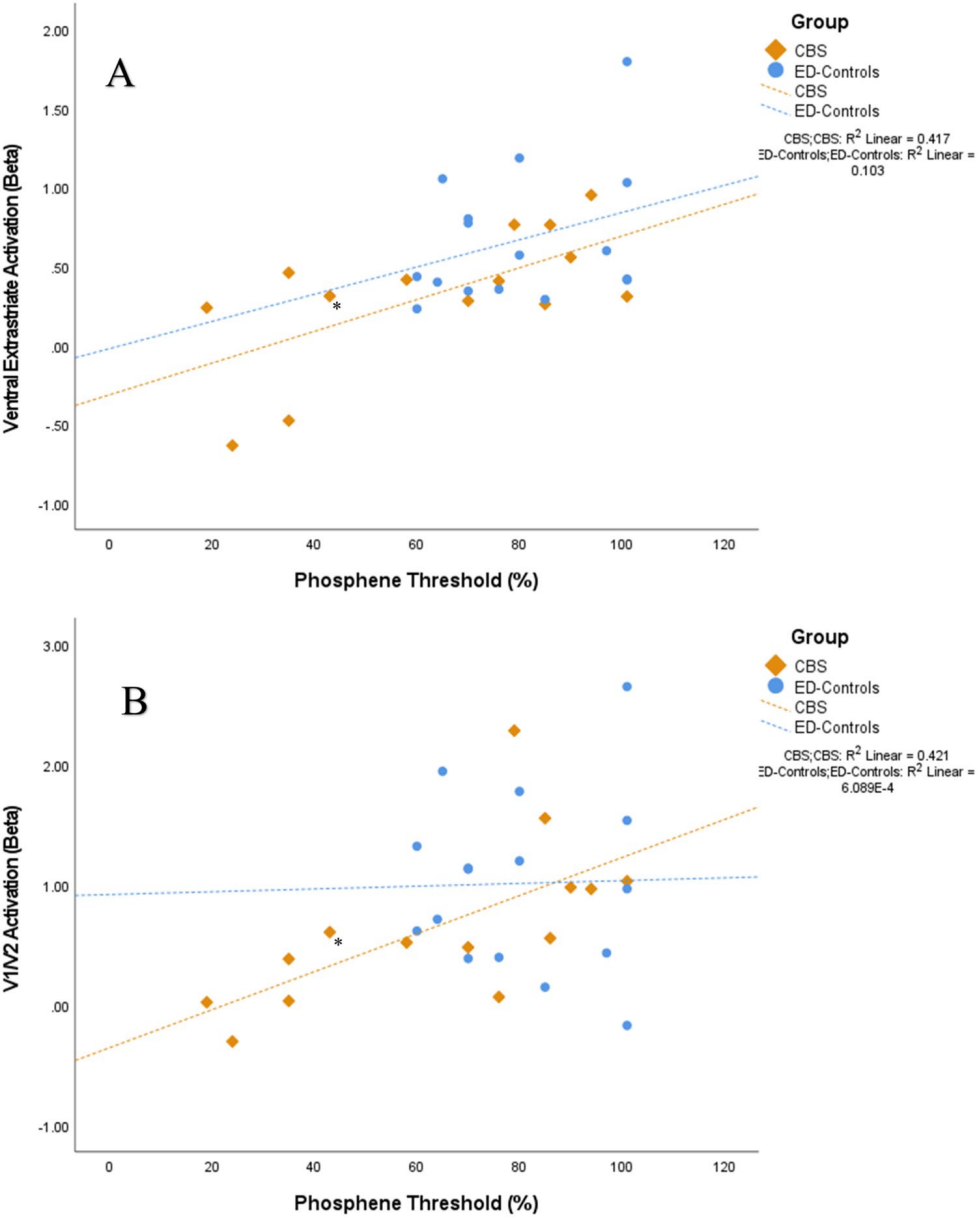
Associations between fMRI BOLD activation and phosphene thresholds in **A** ventral extrastriate and **B** V1/V2, split by group. Lines of best fit calculated from the mean (*R*^2^). * denotes participant with continuous hallucinations (*n* = 1)

A positive relationship between phosphene thresholds with both V1/V2 BOLD activity (*r*_s_ = 0.378, *p* = 0.043) and ventral extrastriate (*r*_s_ = 0.517, *p* = 0.004) BOLD activity was observed across both CBS and ED-controls when controlling for age. Separate analysis of each group indicated a strong relationship between V1/V2 activation and phosphene thresholds in the CBS group specifically (*r*_*s*_ = 0.649, *p* = 0.016) with lower phosphene thresholds associated with lower BOLD activation in these regions (Fig. 5), but no significant relationship in ED-controls (*r*_*s*_ = 0.024, *p* = 0.931).

Lower phosphene thresholds were associated with lower alpha-reactivity in the CBS group (*r*_s_ = 0.580, *p*_uncorr_ = 0.038), but not ED-controls. There were no significant associations between any of the a priori defined EEG bands power and phosphene thresholds across groups. When split by group, lower phosphene thresholds were associated with lower alpha-reactivity in the CBS group (*r*_s_ = 0.580, *p* = 0.038), but not ED-controls. Lower phosphene thresholds were also found to be significantly associated with higher occipital delta (*r*_s_ = −0.908, *p*_uncorr_ < 0.0001) and beta power (*r*_s_ = −0.682, *p*_uncorr_ = 0.01), but lower pre-alpha (*r*_s_ = 0.554, *p*_uncorr_ = *0.0*5) in the CBS group, but not ED-controls. However, only the association with delta power remained following multiple comparison correction. When split by hallucination type, a strong significant relationship between lower phosphene thresholds and alpha power was also observed in simple (*r*_s_ = 0.821, *p* = 0.023) but not complex hallucinators (*r*_s_ = 0.029, *p* = 0.957).

No significant relationships between fMRI BOLD activation and EEG oscillations in any of the groups were noted when corrected for multiple comparisons.

## Discussion

Using a multimodal approach, this study demonstrated altered visual cortical activity specific to CBS, relative to people with eye disease without VH, including reduced primary visual cortex and ventral extrastriate activation in response to visual stimulation, decreased occipital alpha power and alpha-reactivity, and greater variability in phosphene thresholds. This supports the supposition that VH in this group are, at least in part, the result of increased excitability in the visual cortex following sight loss.

### TMS

A trend toward lower phosphene thresholds (indicating greater visual cortical excitability) was apparent in the CBS group, although this did not reach significance when compared to the ED-controls. As even short-term visual deprivation is observed to reduce phosphene thresholds in sighted people [10] it is perhaps unsurprising that both groups, who experience comparative levels of visual impairment, presented with equivalent occipital excitability. Similarly, Taylor and colleagues [26] noted a lack of difference in phosphene thresholds between aged-controls and dementia with Lewy bodies (DLB) hallucinators. Since high inter-individual variability in phosphene thresholds within the general population has previously been noted [31], it is possible that the ED-controls in this study reside on the lower end of the ‘normal’ visual cortical excitability spectrum and are relatively protected against VH following sight loss. Additionally, the observation of significantly greater variability in the CBS group may indirectly indicate greater within-subject instability. This variability would be consistent with dynamic models of episodic VH (e.g. [32, 33]) which emphasise threshold effects. Corresponding instability has been observed in migraineurs with aura compared to those without [34].

Although no differences were observed between groups, the finding that phosphene thresholds were significantly associated with severity in the VH group may further support the potential role of visual cortical hyperexcitability in CBS. The significant negative relationship between phosphene thresholds, VH severity, and irritation indicate that individuals with greater cortical excitability were prone to more severe VH. This is directly comparable to the findings of Taylor et al. [26] who similarly noted that occipital hyperexcitability was strongly correlated with the VH severity in DLB. It is therefore possible that, while hyperexcitability alone may not be directly responsible for VH, it may provide a specific trait-related marker that moderates VH severity.

### fMRI

Functional activation of the occipital cortex in response to simple eye-movements has been reliably reported in previous studies (e.g. [45]). While whole-brain fMRI BOLD activation was comparable across groups, CBS participants demonstrated significantly reduced activity within ventral extrastriate and primary visual cortex compared to ED-controls. Upon closer inspection, this difference was driven almost entirely by complex hallucinators, with simple hallucinators demonstrating more comparable activity to that of ED-controls. In accordance with the present study, ffytche et al. [6] similarly observed reduced BOLD activation in CBS participants related to ongoing ‘tonic’ activity during the ‘OFF’ phase of the stimulus which reduced the difference in BOLD between stimulation and rest. It is possible that the pattern of activity observed in this study may indicate the same trait changes to cortical activity in CBS. Unlike TMS, however, no associations were noted between VH ratings and fMRI BOLD activation, suggesting that, outside periods of active hallucination, changes in BOLD activation from rest may not provide useful measures of the temporal dynamics or severity of VH, even if the location of such changes are associated with the type of hallucination that people experience [6, 12].

### EEG

Congruent with the deafferentation hypothesis, a pattern of decreased eyes-closed occipital alpha-power was noted in the CBS group compared to controls, although this only reached statistical significance when looking at complex hallucinators in comparison to simple hallucinators and ED-Controls. Occipital alpha-power has been posited as a proxy, ‘inverse’ measure of active cortical processing and excitability, with reduced alpha-power associated with increased excitability and visual attention [8, 35]. A similar finding has previously been observed in CBS patients [7].

The analysis of the power spectral density (PSD) observed a notable shift toward lower frequency band oscillations in CBS compared to ED-Controls which, again, appeared to be driven predominantly by complex hallucinators compared to simple. Cortical slowing, posited to be indicative of overall cortical dysfunction, has been observed previously in CBS case reports in posterior and occipital regions [14, 36]. Like the present study, increased posterior theta activity has also been observed in CBS [37, 38].

The patterns of cortical slowing, particularly with shifts towards the theta band, and reductions in alpha activity are often observed in healthy aging populations [39]. However, as the groups in this study were matched for age, these differences are likely due to more than physiological aging alone. Indeed, the increased theta activity has been found to play a key role in the formation of hallucinations in people with schizophrenia [40] and has been noted in people with VH associated with Alzheimer’s disease, DLB, and epilepsy [41–43].

An increased theta/alpha ratio was observed in CBS complex hallucinators compared to both simple hallucinators and ED-Controls in this study. The Theta/alpha ratio has been posited as a reliable, indirect, marker of cholinergic dysfunction in other hallucinating pathologies [43, 44] with increased ratios significantly associated with both visual spatial dysfunction and risk of VH in Alzheimer’s disease and Lewy body disease patients [43]. While it is still unclear how changes toward slow-wave activity may be directly related to VH in CBS, it is possible that cholinergic dysfunction may contribute to the formation of complex VH in this group, warranting further investigation utilising more direct measures of cholinergic activity (e.g. PET-MR cholinergic tracers). Nevertheless, it is possible that alterations in cholinergic activity produce a neurophysiological brain state and functional changes in the early visual cortex and association cortices which, alongside reduction of visual input through eye disease, may be necessary for CBS to occur.

### Multi-modal analysis

Comparable to the findings of ffytche et al. [6], lower task-related differential activity in the visual cortex was observed in the CBS group that was thought to reflect increased activity during rest. This would account for the positive association found in this study between BOLD activation and EEG alpha-reactivity, particularly in the CBS group: visual cortex is less responsive to visual stimulation or eye closure in CBS than in ED-Controls because it is active during both rest and visual stimulation. The findings are also consistent with the association between lower phosphene thresholds with lower activation in the ventral extrastriate cortex and V1/V2, and lower alpha-reactivity in the CBS group but not ED-controls. Taken together, the findings are consistent with predictions of the deafferentation hypothesis with the CBS group having continuously active visual cortex which, as a result, is less responsive to visual stimulation from eye movements, does not reduce activity with eye closure, and is more sensitive to direct excitatory stimulation (e.g. TMS). The implication is that people who experience CBS possess a visual cortex in which spontaneous internally generated activity as a consequence of sight loss results in hallucinations. This proposal is consistent with a recent study in CBS that posited that hallucinations arise due to a slow build-up of neural activity in early visual regions, alongside a greater susceptibility to spontaneous noise fluctuations [46].

In further support of the increased cortical excitability account of our findings, a treatment trial performed in the same CBS cohort investigating the efficacy of inhibitory non-invasive brain stimulation of the primary visual cortex found that participants who demonstrated reduced occipital alpha-power and alpha-reactivity prior to stimulation were more likely to report a positive therapeutic response to stimulation, in the form of reduced VH frequency, indicating that patients with higher occipital excitability may benefit more from inhibitory brain stimulation[18].

#### Simple vs complex hallucinations

Across approaches it was consistently observed that differences in visual cortical activity between CBS and ED-Controls were predominantly driven by complex hallucinators, while simple hallucinators demonstrated cortical activation more closely aligned to that of ED-controls. While previous studies have postulated differences in the spatial dynamics of cortical activity influencing VH phenomenology (e.g. [3]), the present study did not note any significant differences in cortical BOLD activation at any of the ROIs investigated between simple and complex hallucinators. However, it was noted that complex hallucinators demonstrated stronger evidence of increased excitability in the early visual cortex in response to TMS, decreased BOLD activation in response to visual stimulation, and increased occipital slowing than simple hallucinators. Bayesian principles suggest that prior knowledge and expectations of the perceiver are crucial to determining accurate signal detection [47]. It is, therefore, possible that sensory ambiguity in CBS due to reduced visual input may create an environment in which spontaneous bottom-up activity is influenced by previous sensory based learning, directing attention and attributing meaning towards internally generated activity and resulting in VH [48]. In the context of hallucination phenomenology, this may explain why altered cortical activity was most prominent in participants with complex rather than simple VH, as prior expectations provide a bias toward complex imagery [33].

The models of VH often separate simple and complex hallucinators and suggest that, while simple hallucinations may occur concurrently with complex VH, these may arise as a result of different mechanisms or from different cortical locations (e.g. [33]). The findings of this study support those suggestions. Another possibility is that the lack of a reliable difference between simple and complex hallucinators (possibly reflecting a lack of statistical power due to the small group sizes) on the majority of measures in the present study, in conjunction with a significant difference between complex hallucinators and ED-controls, may indicate that the simple hallucinators represent an intermediatory group between controls and complex hallucinators. The stratification of CBS into distinct groups (i.e. based on phenomenology) may help future research to not only better interrogate the underlying aetiology of these hallucinations but develop more effective interventions.

#### Future directions

We have previously reported a comparative analysis of structural changes in the visual cortex of the CBS and ED-control group [49] which found no macrostructural or connectivity differences between the two groups. It is, therefore, likely that the results described above are due to altered functional connectivity in CBS compared to ED-controls. Although EEG and fMRI were both performed in this study, EEG was restricted in focus to posterior/occipital regions due to the limited spatial sampling of the combined tDCS-EEG equipment used. Future research could seek to further investigate the role of functional connectivity changes between the visual cortex and more anterior brain regions and their contribution to VH formation in CBS.

Relating changes in hallucinations to variation in the function of visual cortex over time is another potential area of future investigation. In the CBS group, VH were seen to start anywhere from immediately to 19 years after sight loss, implying a critical threshold of deafferentation required before VH onset or a highly variable latency period. It may, therefore, be interesting to study potential longitudinal changes to cortical activity in eye disease, to assess whether changes evidenced in CBS develop over time. As CBS has been reported, in some cases, to be self-limiting [50, 51] this may also indicate that while cortical changes may be an initially adaptive or compensatory response to sight loss, the cortex may habituate over time, lending further support to dynamic models of VH (e.g. [33]). In addition, comparisons to age-matched, healthy individuals with no visual pathology may also provide additional insights into the contribution of neurophysiological changes associated with vision loss to the development of CBS.

### Strengths and limitations

A key strength of the present study was the recruitment of age and visual acuity matched groups both with and without visual hallucinations, allowing for the specific investigation of the mechanisms involved in VH separate to eye disease, and the combination of electrophysiological and imaging measures of visual cortical activity and excitability. Though the study had a small overall sample size, this is not unusual in CBS studies due to the relative rarity of participants with ongoing hallucinations that meet study criteria and this study is relatively large compared to previous neurophysiological or imaging studies of CBS. While CBS has greater prevalence in elderly populations, perhaps due to the increased prevalence of eye disease [50, 51], it has also been observed in younger populations and children [50, 52]. As such, the present findings may not be representative of people with younger onset CBS who may present with a different pattern of altered cortical activity following sight loss.

A further limitation is the way in which visual function was assessed in this study. The measure of visual acuity and contrast was taken across both eyes (binocular acuity sensitive to the best eye), as opposed to each eye individually and the fact that the degree of remaining peripheral vision in each eye was not quantifiably assessed in each participant, means that it is possible that the two groups were not as closely visually matched as suggested by their binocular acuity values. Indeed, the significant difference in IADL scores, with the CBS group reporting significantly worse independence than controls, may be due to differences in vision in the worst eye or peripheral visual field rather than hallucination status. Given that worst eye acuity is likely to be a better index of deafferentation than best eye acuity and the fMRI visual stimulation task was designed to stimulate peripheral vision, it is possible that unidentified differences in visual function may have contributed to some of the differences observed between, and variability within, the groups. Similarly, as phosphene thresholds were not repeat measured across cortical sites, it is difficult to address the potential impact of such within-subject variation.

Finally, the current study is only able to provide evidence towards potential trait differences in cortical activity between people with and without VH in eye disease. As investigations were performed when participants were not actively hallucinating (other than two participants with continuous hallucinations), it is not possible to conclude how changes to activity develop during the hallucination state. Nevertheless, this is a pervasive limitation in hallucination research due to the unpredictable occurrence of VH. However, the integration of cortical measures during both the ON and OFF phase of VH in CBS and their transition in future studies would undoubtedly improve interrogation of their aetiology.

## Conclusion

The present study provides converging lines of evidence for increased visual cortical excitability in CBS compared to eye disease controls without VH. While the visual cortex of people with CBS appears less reactive to visual stimulation caused by eye movements and closure than ED-controls, this is likely to be caused by persistent activation associated with increased occipital excitability which, in turn, was associated with more severe VH. The reason why these functional alterations in response to sight loss occur in some eye disease patients but not others remain unclear. Furthermore, the functional differences found were more pronounced in CBS with predominantly complex hallucinations compared to CBS with predominantly simple hallucinations and may further support the inference that simple and complex VH relate to different mechanisms or cortical locations.

## Supplementary Information

Below is the link to the electronic supplementary material.

Supplementary file1 (PDF 350 KB)

Supplementary file2 (PDF 222 KB)

## Data Availability

The data supporting the findings presented in this paper are available on the basis of a formal data-sharing agreement and, depending on data usage, agreement for formal collaboration, and co-authorship where appropriate.

## References

[1] Teunisse RJ, Cruysberg JR, Hoefnagels WH, Verbeek AL, Zitman FG (1996) Visual hallucinations in psychologically normal people: Charles Bonnet’s syndrome. Lancet 347(9004):794–797 8622335 10.1016/s0140-6736(96)90869-7

[2] Urwyler P, Nef T, Müri R, Archibald N, Makin SM, Collerton D, et al (2013) Determination of the dielectric tensor function of triclinic CuSO4·5H2O. Vib Spectrosc. 2013

[3] Ffytche DH (2007) Visual hallucinatory syndromes: past, present, and future. Dialogues Clin Neurosci 9(2):173–189 17726916 10.31887/DCNS.2007.9.2/dffytche PMC3181850

[4] Cogan DG (1973) Visual hallucinations as release phenomena. Albrecht Von Graefes Arch Klin Exp Ophthalmol 188(2):139–150 4543235 10.1007/BF00407835

[5] Burke W (2002) The neural basis of Charles Bonnet hallucinations: a hypothesis. J Neurol Neurosurg Psychiatr 73:535–541 10.1136/jnnp.73.5.535 PMC1738134 12397147

[6] Ffytche DH, Howard R, Brammer M, David A, Woodruff P, Williams S (1998) The anatomy of conscious vision: an fMRI study of visual hallucinations. Nat Neurosci 1(8):738–742 10196592 10.1038/3738

[7] Hanoglu L, Yildiz S, Polat B, Demirci S, Tavli AM, Yilmaz N et al (2016) Therapeutic effects of rivastigmine and alfa-lipoic acid combination in the Charles Bonnet syndrome: electroencephalography correlates. Curr Clin Pharmacol 11:270–273 27697039 10.2174/1574884711666161003153616

[8] Thut G, Nietzel A, Brandt S, Pascual-Leone A (2006) Alpha-band electroencephalographic activity over occipital cortex indexes visuospatial attention bias and predicts visual target detection. J Neurosci 26(37):9494–9502. 10.1523/JNEUROSCI.0875-06.2006 16971533 10.1523/JNEUROSCI.0875-06.2006 PMC6674607

[9] Painter DR, Dwyer MF, Kamke MR, Mattingley JB (2018) Stimulus-driven cortical hyperexcitability in individuals with Charles Bonnet hallucinations. Curr Biol 28(21):3475-3480.e3. 10.1016/j.cub.2018.08.058 30415703 10.1016/j.cub.2018.08.058

[10] Boroojerdi B (2000) Enhanced excitability of the human visual cortex induced by short-term light deprivation. Cereb Cortex 10(5):529–534 10847602 10.1093/cercor/10.5.529

[11] Sireteanu R, Oertel V, Mohr H, Linden D, Singer W (2008) Graphical illustration and functional neuroimaging of visual hallucinations during prolonged blindfolding: a comparison to visual imagery. Perception 37(12):1805–1821 19227374 10.1068/p6034

[12] Adachi N, Watanabe T, Matsuda H, Onuma T (2000) Hyperperfusion in the lateral temporal cortex, the striatum and the thalamus during complex visual hallucinations: single photon emission computed tomography findings in patients with Charles Bonnet Syndrome. Psychiatr Clin Neurosci 54:157–162 10.1046/j.1440-1819.2000.00652.x 10803809

[13] Kazui H, Ishii R, Yoshida T, Ikezawa K, Takaya M, Tokunaga H et al (2009) Neuroimaging studies in patients with Charles Bonnet Syndrome. Psychogeriatrics 9(2):77–84 19604330 10.1111/j.1479-8301.2009.00288.x

[14] Ossola M, Romani A, Tavazzi E, Pichiecchio A, Galimberti CA (2010) Epileptic mechanisms in Charles Bonnet syndrome. Epilepsy Behav 18(1–2):119–122. 10.1016/j.yebeh.2010.03.010 20471325 10.1016/j.yebeh.2010.03.010

[15] Nuzzi R, Dallorto L, Vitale A (2020) Cerebral modifications and visual pathway reorganization in maculopathy: a systematic review. Vol. 14, Frontiers in Neuroscience. Frontiers Media S.A 10.3389/fnins.2020.00755 PMC7472840 32973424

[16] Boucard CC, Hernowo AT, Maguire RP, Jansonius NM, Roerdink JBTM, Hooymans JMM et al (2009) Changes in cortical grey matter density associated with long-standing retinal visual field defects. Brain 132(Pt 7):1898–1906 19467992 10.1093/brain/awp119 PMC2702836

[17] Ffytche DH, Blom JD, Catani M (2010) Disorders of visual perception. J Neurol Neurosurg Psychiatry Vol. 81:1280–1287 10.1136/jnnp.2008.171348 20972204

[18] daSilva MK, Schumacher J, Collerton D, Colloby S, Elder GJ, Olsen K et al (2022) Transcranial direct current stimulation in the treatment of visual hallucinations in Charles Bonnet syndrome: a randomized placebo-controlled crossover trial. Ophthalmology 129(12):1368–1379 35817197 10.1016/j.ophtha.2022.06.041

[19] Reischies FM, Geiselmann B (1997) Age-related cognitive decline and vision impairment affecting the detection of dementia syndrome in old age. Brit J Psychiatr 171:449–451 10.1192/bjp.171.5.449 9463604

[20] Cummings JL, Mega M, Gray K, Rosenberg-Thompson S, Carusi DA, Gornbein J (1994) The neuropsychiatric inventory. Neurology 44(12):2308 LP – 2308 7991117 10.1212/wnl.44.12.2308

[21] Mosimann UP, Collerton D, Dudley R, Meyer TD, Graham G, Dean JL et al (2008) A semi-structured interview to assess visual hallucinations in older people. Int J Geriatr Psychiatry 23:712–718 18181237 10.1002/gps.1965

[22] Yesavage JA, Brink TL, Rose TL, Lum O, Huang V, Adey M et al (1982) Development and validation of a geriatric depression screening scale: a preliminary report. J Psychiatr Res 17(1):37–49 7183759 10.1016/0022-3956(82)90033-4

[23] Lawton M, Brody E (1969) Assessment of older people: self-maintaining and instrumental activities of daily living. Gerontologist 9(3):179–186 5349366

[24] Bach M (2007) The Freiburg visual acuity test-variability unchanged by post-hoc re-analysis. Graefe’s Arch Clin Exp Ophthalmol 245(7):965–971 17219125 10.1007/s00417-006-0474-4

[25] Sparing R, Dambeck N, Stock K, Meister IG, Huetter D, Boroojerdi B (2005) Investigation of the primary visual cortex using short-interval paired-pulse transcranial magnetic stimulation (TMS). Neurosci Lett 382(3):312–316 15925110 10.1016/j.neulet.2005.03.036

[26] Taylor JP, Firbank M, Barnett N, Pearce S, Livingstone A, Mosimann U et al (2011) Visual hallucinations in dementia with Lewy bodies: transcranial magnetic stimulation study. Br J Psychiatry 199(6):492–500 22016436 10.1192/bjp.bp.110.090373 PMC3227808

[27] Rottschy C, Eickhoff SB, Schleicher A, Mohlberg H, Kujovic M, Zilles K et al (2007) Ventral visual cortex in humans: cytoarchitectonic mapping of two extrastriate areas. Hum Brain Mapp 28(10):1045–1059 17266106 10.1002/hbm.20348 PMC6871378

[28] Eickhoff S, Stephan K, Mohlberg H, Grefkes C, Fink G, Amunts K et al (2005) A new SPM toolbox for combining probabilistic cytoarchitectonic maps and functional imaging data. Neuroimage 25(4):1325–1335 15850749 10.1016/j.neuroimage.2004.12.034

[29] Wan L, Huang H, Schwab N, Tanner J, Rajan A, Lam NB et al (2019) From eyes-closed to eyes-open: role of cholinergic projections in EC-to-EO alpha reactivity revealed by combining EEG and MRI. Hum Brain Mapp 40(2):566–577 30251753 10.1002/hbm.24395 PMC6338213

[30] Schmidt MT, Kanda PAM, Basile LFH, da Silva Lopes HF, Baratho R, Demario JLC et al (2013) Index of alpha/theta ratio of the electroencephalogram: a new marker for Alzheimer’s disease. Front Aging Neurosci 5:60 24130529 10.3389/fnagi.2013.00060 PMC3793211

[31] Kammer T (1998) Phosphenes and transient scotomas induced by magnetic stimulation of the occipital lobe: their topographic relationship. Neuropsychologia 37(2):191–198 10.1016/s0028-3932(98)00093-1 10080376

[32] Tsukada H, Fujii H, Aihara K, Tsuda I (2015) Computational model of visual hallucination in dementia with Lewy bodies. Neural Netw 62(1):73–82 25282547 10.1016/j.neunet.2014.09.001

[33] Collerton D, Barnes J, Diederich NJ, Dudley R, ffytche D, Friston K, et al. (2023) Understanding visual hallucinations: a new synthesis. Vol. 150, Neuroscience and Biobehavioral Reviews. Elsevier Ltd 10.1016/j.neubiorev.2023.105208 37141962

[34] Aurora S, Cao Y, Bowyer SM, Welch KMA (1999) The occipital cortex is hyperexcitable in migraine: experimental evidence. Headache 39(7):469–476 11279929 10.1046/j.1526-4610.1999.3907469.x

[35] Barry RJ, Clarke AR, Johnstone SJ, Magee CA, Rushby JA (2007) EEG differences between eyes-closed and eyes-open resting conditions. Clin Neurophysiol 118(12):2765–2773 17911042 10.1016/j.clinph.2007.07.028

[36] Josephson SA, Kirsch HE (2006) Complex visual hallucinations as post-ictal cortical release phenomena. Neurocase 12(2):107–110 16714243 10.1080/13554790500519722

[37] Piarulli A, Annen J, Kupers R, Laureys S, Martial C (2021) High-density eeg in a charles bonnet syndrome patient during and without visual hallucinations: a case-report study. Cells 10(8):1991 34440760 10.3390/cells10081991 PMC8392863

[38] Yildiz S, Yulug B, Kocabora MS, Hanoglu L (2021) Power spectral density and coherence analysis of eye disease with and without visual hallucination. Neurosci Lett 1:740 10.1016/j.neulet.2020.135444 33127444

[39] Ishii R, Canuet L, Aoki Y, Hata M, Iwase M, Ikeda S et al (2017) Healthy and pathological brain aging: from the perspective of oscillations, functional connectivity, and signal complexity. Neuropsychobiology 75(4):151–161 29466802 10.1159/000486870

[40] Boutros NN, Arfken C, Galderisi S, Warrick J, Pratt G, Iacono W (2008) The status of spectral EEG abnormality as a diagnostic test for schizophrenia. Schizophrenia Res 99:225–237 10.1016/j.schres.2007.11.020 PMC2288752 18160260

[41] Lopez OL, Becker JT, Brenner RP, Rosen J, Bajulaiye OI, Reynolds CF (1991) Alzheimer’s disease with delusions and hallucinations: neuropsychological and electroencephalographic correlates. Neurology 41(6):906–912 2046938 10.1212/wnl.41.6.906

[42] Oishi M, Otsubo H, Kameyama S, Wachi M, Tanaka K, Masuda H et al (2003) Ictal magnetoencephalographic discharges from elementary visual hallucinations of status epilepticus. J Neurol Neurosurg Psychiatr 74(4):525–527 10.1136/jnnp.74.4.525 PMC1738400 12640082

[43] Baik K, Jung JH, Jeong SH, Chung SJ, Yoo HS, Lee PH et al (2022) Implication of EEG theta/alpha and theta/beta ratio in Alzheimer’s and Lewy body disease. Sci Rep. 10.1038/s41598-022-21951-5 36333386 10.1038/s41598-022-21951-5 PMC9636216

[44] Bonanni L, Thomas A, Tiraboschi P, Perfetti B, Varanese S, Onofrj M (2008) EEG comparisons in early Alzheimer’s disease, dementia with Lewy bodies and Parkinson’s disease with dementia patients with a 2-year follow-up. Brain 131(3):690–705 18202105 10.1093/brain/awm322

[45] Bodis-Wollner I, Tzelepi A, Sagliocco L, Bandini F, Mari Z, Pierantozzi M et al (1997) Visual processing deficit in Parkinson’s disease. Brain Topography Today 1147:606–611

[46] Hahamy A, Wilf M, Rosin B, Behrmann M, Malach R (2021) How do the blind “see”? The role of spontaneous brain activity in self-generated perception. Brain 144(1):340–353 33367630 10.1093/brain/awaa384 PMC7880672

[47] Horga G, Abi-Dargham A (2019) An integrative framework for perceptual disturbances in psychosis. Nat Rev Neurosci 20(12):763–778. 10.1038/s41583-019-0234-1 31712782 10.1038/s41583-019-0234-1

[48] Collerton D, Perry E, McKeith I (2005) Why people see things that are not there: a novel perception and attention deficit model for recurrent complex visual hallucinations. Behavioral Brain Sci 28(6):737–757 10.1017/S0140525X05000130 16372931

[49] Firbank MJ, daSilva MK, Collerton D, Elder GJ, Parikh J, Olsen K et al (2022) Investigation of structural brain changes in Charles Bonnet Syndrome. Neuroimage Clin 1:35 10.1016/j.nicl.2022.103041 PMC9118504 35576854

[50] Menon GJ, Rahman I, Menon SJ, Dutton GN (2003) Complex visual hallucinations in the visually impaired: the Charles Bonnet syndrome. Surv Ophthalmol 48(1):58–72 12559327 10.1016/s0039-6257(02)00414-9

[51] Khan JC, Shahid H, Thurlby DA, Yates JRW, Moore AT (2008) Charles Bonnet syndrome in age-related macular degeneration: The nature and frequency of images in subjects with end-stage disease. Ophthalmic Epidemiol 15(3):202–208 18569816 10.1080/09286580801939320

[52] Moosajee M, Jones L (2020) Visual hallucinations and sight loss in children and young adults: a retrospective case series of Charles Bonnet syndrome. Brt J Opthalmol. 10.1136/bjophthalmol-2020-317237 10.1136/bjophthalmol-2020-317237 PMC8543192 32933935

