# Supplementary material for: Visual cortical activity in Charles Bonnet syndrome: testing the deafferentation hypothesis
Source: J Neurol. 2025 Feb 11;272(3):199. doi: 10.1007/s00415-024-12741-2 (PMC11813974; doi:10.1007/s00415-024-12741-2)
Supplement: Supplementary file 1 — Supplementary file1 (PDF 350 KB) [file 415_2024_12741_MOESM1_ESM.pdf]

Visual Cortical Activity in Charles Bonnet Syndrome: Testing the Deafferentation Hypothesis. Journal of Neurology. daSilva Morgan, K*; Collerton, D; Firbank, MJ; Schumacher, J; ffytche, DH; Taylor, J-P .
*corresponding author: Kat.da-silva-morgan@newcastle.ac.uk Translational and Clinical Research Institute, Campus for Ageing and Vitality, Newcastle upon Tyne, NE4 5PL, United Kingdom

Online Resource 1.

Supplementary materials: fMRI task

People with macular disease have loss of vision in the central visual field but sparing of vision in the peripheral visual field. This poses difficulties for fMRI studies of the cortical visual system in macular conditions as it impairs the ability to establish and maintain stable fixation. To address this issue, a Macular Society funded grant (Non-drug treatments for visual hallucinations) included development of a visual stimulus for use in macular disease that did not require stable fixation. The proof-of-concept study explored whether the peripheral visual field in people with age-related macular disease could be stimulated by shifting gaze across a high contrast checkerboard pattern (checks ~3˚) as well as two factors that might influence the visual cortical response: visual contrast boundaries in the stimulus and the rate of gaze shift.

Participants were asked move their gaze from left to right across the entire horizontal extent of stimulus backgrounds that varied in their contrast content (a checkerboard or a homogenous grey blank screen) at two frequencies of gaze shift (one shift every second or one shift every 3 seconds) in a factorial design. Gaze shifts were initiated by a computer-generated voice prompt. In the control condition participants looked straight ahead at the grey blank screen with a voice prompt 'rest' every three seconds. Each condition was presented for 15 seconds and repeated 4 times in pseudorandom order (total task duration 5 minutes). Eye movements were monitored using a video camera mounted in the stimulus presentation goggles (Nordic Neurolab Visual system with in-built eye tracking).

The task was piloted in 23 participants with age-related macular disease (13 with CBS and 10 ED-controls; mean age CBS 79.9±5.5 years). Figure S1 shows a representative eye tracking record demonstrating that participants were able to carry out the task and maintain the same amplitude of gaze-shift over the course of 5 minutes. The trajectory of left-right eye movement is approximately 30˚ of visual angle; however, it was not possible to calibrate this for each participant because of the difficulty in establishing a stable fixation point.

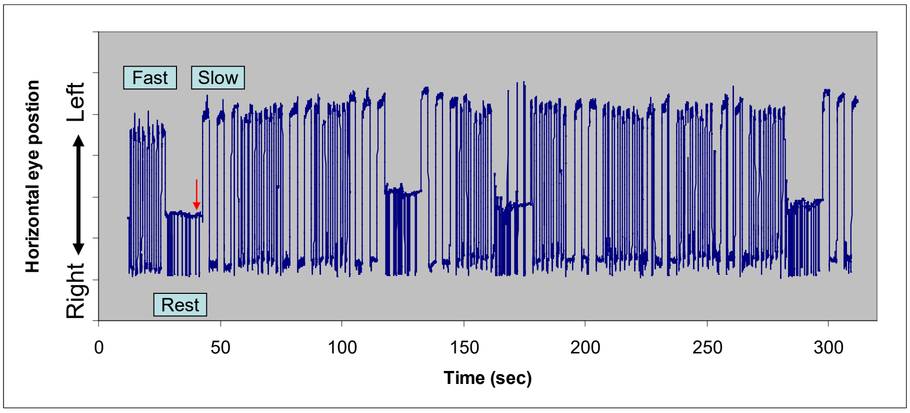

Figure S1 Eye-movement recording Data from a representative participant. Randomised blocks of gaze shifts every second (marked Fast) and gaze shifts every three seconds (marked Slow) are shown interspersed with control periods in which the subject looked straight ahead (marked Rest). The spike-like deflections in the rest block are caused by blinks (example marked by red arrow).

Figure S2 shows occipital activations for the four gaze shift conditions compared to rest in the CBS group. The occipital lobe was activated in each condition, most prominently in the region mapping the peripheral visual field. Maximal activation of the occipital lobe occurred for the checkerboard with gaze shift every second condition.

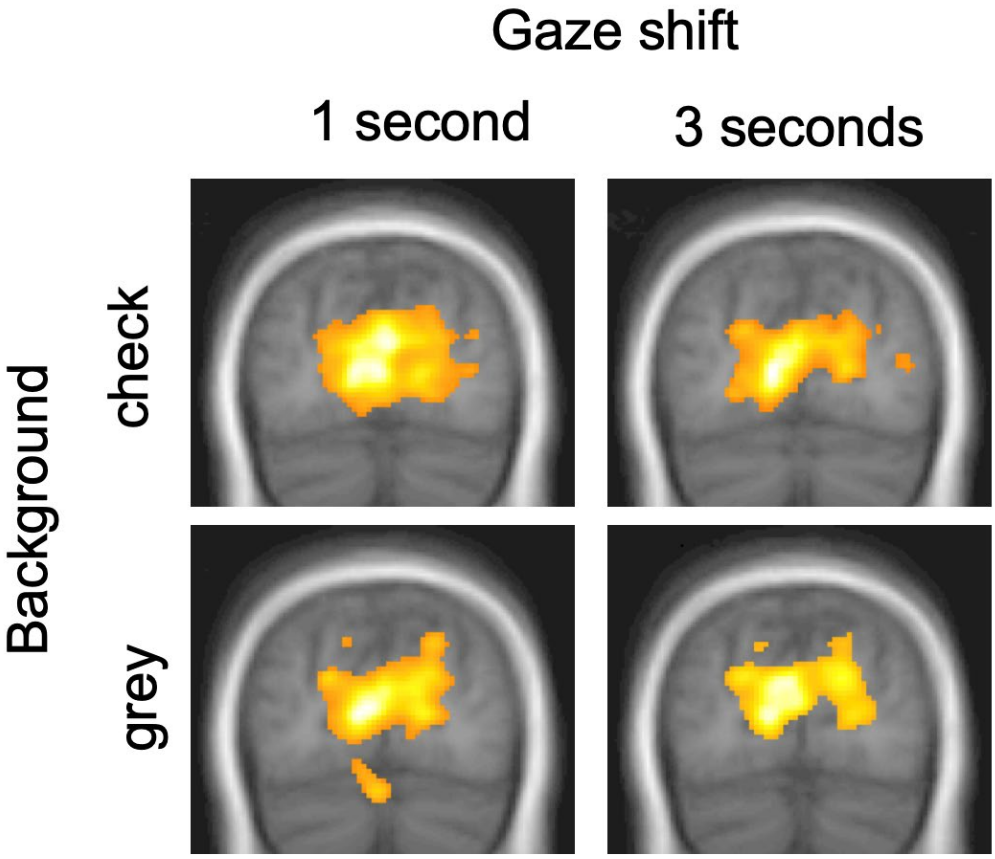

Figure S2 Occipital activation The CBS group activation for each of the four conditions (checkerboard | gaze shift 1 sec; checkerboard | gaze shift 3 sec; grey | gaze shift 1 sec; grey | gaze shift 3 sec) compared to the rest condition. A single coronal slice is shown through the occipital lobe (threshold $p<0.001$ uncorrected).

Adaptation of stimulus for current study

The feasibility study informed the design of the stimulus for the current study. Instead of repeating the factorial design methodology, we focussed on the stimulus condition with maximal occipital activation (checkerboard background | gaze shift every second) and compared this to the rest condition. As we had used eye tracking in the feasibility study and confirmed participants with age-related macular disease were able to carry out the task, we did not repeat this for the current study.
