# Supplementary material for: Visual cortical activity in Charles Bonnet syndrome: testing the deafferentation hypothesis
Source: J Neurol. 2025 Feb 11;272(3):199. doi: 10.1007/s00415-024-12741-2 (PMC11813974; doi:10.1007/s00415-024-12741-2)
Supplement: Supplementary file 2 — Supplementary file2 (PDF 222 KB) [file 415_2024_12741_MOESM2_ESM.pdf]

Visual Cortical Activity in Charles Bonnet Syndrome: Testing the Deafferentation Hypothesis. Journal of Neurology. daSilva Morgan, K*; Collerton, D; Firbank, MJ; Schumacher, J; ffytche, DH; Taylor, J-P .

*corresponding author: Kat.da-silva-morgan@newcastle.ac.uk Translational and Clinical Research Institute, Campus for Ageing and Vitality, Newcastle upon Tyne, NE4 5PL, United Kingdom

Online Resource 2.

Summary of visual hallucination (VH) features reported by the Charles Bonnet Syndrome (CBS) group, including predominant hallucination phenomenology (sub-group), frequency, duration and emotional impact as rated on the North East Visual Hallucinations Interview (NEVHI) and examples of reported phenomenology. Emotional impact was categories by average NEVHI irritation and distress scores: 0-3 = low, 4-7= moderate, 8-10 = high. Multiple VH a day/week indicates three or more VH during this time period.

PARTICIPANT	AGE	PRIMARY EYE DISEASE	PREDOMINANT HALLUCINATION PHENOMENOLOGY	YEARS OF CBS	FREQUENCY	DURATION	EMOTIONAL IMPACT	EXAMPLE PHENOMENOLOGY
**1**	75	Macular Degeneration	Complex	5	Continuous while awake	Continuous while awake	Moderate	Rapidly spinning pinwheel; moving complex patterns; coloured 'fireworks'
**2**	75	Advanced Chloroquine retinopathy	Complex	13	Multiple times a day	Hours	Moderate	Panoramic scene of destroyed buildings; wallpaper patterns; orange lines
**3**	73	Macular Degeneration	Simple	1	Multiple times a day	Seconds – Minutes	Moderate	Starbursts of moving lights; spinning cylindrical lights
**4**	80	Macular Degeneration	Complex	1	Multiple Times a Week	Seconds-minutes	Low	Small turtles/zebras running across the floor; circular and square flashing lights
**5**	85	Macular Degeneration	Complex	3	Multiple times a day	Hours	Moderate	'Parquet flooring' pattern; houses and fences; bright sparkling lights
**6**	89	Macular Degeneration	Complex	4	Multiple times a week	Seconds - minutes	Moderate	Parked and moving vehicles; people standing outside of the window
**7**	93	Macular Degeneration	Complex	1	Multiple times a week	Minutes	Low	Blue and pink lace patterns; an 'ape' sitting in the garden.
**8**	79	Glaucoma	Simple	2	Continuous while awake	Continuous while awake	High	Raindrops hitting a windscreen at high speed; Occasional black and white paisley patterns

**9**	86	Macular Degeneration	Complex	4	Multiple times a week	Minutes	Moderate	Chessboard patterns; realistic faces and queues or crowds of figures; houses and fences
**10**	53	Chronic Uveitis	Complex	2	Multiple times a day	Minutes	Moderate	Moving complex ‘spikey’ shapes; vibrant coloured lights; detailed faces (i.e. ‘Hindu gods’)
**11**	85	Macular Degeneration	Complex	1	Multiple times a week	Seconds - minutes	Low	Netting patterns covering everything in vision; Black amorphous shapes ‘like soot’
**12**	83	Macular Degeneration	Simple	2	Multiple times a week	Minutes	Moderate	Flashing coloured lights; moving/growing black square; mesh patterns
**13**	76	Macular Degeneration	Simple	3	Multiple times a day	Seconds - hours	Low	Blue flashing light; yellow spiderwebs
**14**	87	Macular Degeneration	Complex	2	Multiple times a day	Minutes	Low	Scenes of towns/countryside; scrap metal piles
**15**	71	Glaucoma	Simple	1	Multiple times a day	Minutes – hours	High	Circular/oval/rectangle flashing silver lights
**16**	68	Retinitis Pigmentosa	Simple	10	Multiple times a day	Seconds - minutes	High	White and Blue spinning ‘boomerangs’.
**17**	92	Macular Degeneration	Simple	3	Multiple times a day	Hours	High	Oblong blue shapes; Dark moving blobs; Gargoyle faces
**18**	79	Macular Degeneration	Simple	1	Multiple times a day	Seconds minutes	Low	Oval shape filled with brightly coloured dots and circles
**19**	67	Glaucoma	Complex	3	Multiple times a day	Minutes- Hours	Moderate	‘William Morris Wallpaper’; Coloured mosaic tiles; kaleidoscopic colours
